# Systematic Construction and Validation of a Novel Macrophage Differentiation–Associated Prognostic Model for Clear Cell Renal Cell Carcinoma

**DOI:** 10.3389/fgene.2022.877656

**Published:** 2022-06-14

**Authors:** Chen Liu, Xuhui Zhang, Caoyang Hu, Xuezhi Liang, Xiaoming Cao, Dongwen Wang

**Affiliations:** ^1^ First Clinical Medical College, Shanxi Medical University, Taiyuan, China; ^2^ Department of Urology, First Hospital of Shanxi Medical University, Taiyuan, China; ^3^ Department of Urology, Cancer Hospital Shenzhen Hospital, Chinese Academy of Medical Sciences and Peking Union Medical College, Shenzhen, China

**Keywords:** clear cell renal cell carcinoma, macrophage differentiation, prognostic, TCGA, bioinformatics

## Abstract

**Background:** Clear cell renal cell carcinoma (ccRCC) is a malignant tumor of the human urinary system. Macrophage differentiation is associated with tumorigenesis. Therefore, exploring the prognostic value of macrophage differentiation–associated genes (MDGs) may contribute to better clinical management of ccRCC patients.

**Methods:** The RNA sequence data of ccRCC were obtained from The Cancer Genome Atlas (TCGA) database. Differentially expressed MDGs were unveiled in ccRCC and normal samples. The prognostic model was established according to the univariate and multivariate Cox regression analyses. By combining clinico-pathological features and prognostic genes, a nomogram was established to predict individual survival probability. The Tumor Immune Estimation Resource (TIMER) database was utilized to analyze the correlation between prognostic genes and immune infiltrating cells. Eventually, the mRNA and protein expression levels of prognostic genes were verified.

**Results:** A total of 52 differentially expressed prognosis-related MDGs were identified in ccRCC. Afterward, a six-gene prognostic model (ABCG1, KDF1, KITLG, TGFA, HAVCR2, and CD14) was constructed through the Cox analysis. The overall survival in the high-risk group was relatively poor. Moreover, the risk score was identified as an independent prognostic factor. We constructed a prognostic nomogram with a well-fitted calibration curve based on risk score and clinical data. Furthermore, the prognostic genes were significantly related to the level of immune cell infiltration including B cells, CD8+T cells, CD4+T cells, macrophages, neutrophils, and dendritic cells. Finally, the mRNA expression of prognostic genes in clinical ccRCC tissues showed that the ABCG1, HAVCR2, CD14, and TGFA mRNA in tumor samples were increased compared with the adjacent control tissue samples, while KDF1 and KITLG were decreased, which was consistent with the verification results in the GSE53757.

**Conclusion:** In conclusion, this study identified and validated a macrophage differentiation–associated prognostic model for ccRCC that could be used to predict the outcomes of the ccRCC patients.

## Introduction

Renal cell carcinoma (RCC) is the most common malignant tumor in the kidney, accounting for about 2%–3% of all cancers (Wang et al., 2019). Clear cell renal cell carcinoma (ccRCC) is the most common pathological subtype of RCC. It originates from the proximal ureter and shows certain invasiveness (Liang et al., 2020). ccRCC has a high incidence rate and mortality rate and poor prognosis. Although tumor detection and treatment methods have made some progress and the survival rate of ccRCC patients has also been significantly improved, the overall survival and progression-free survival are still low (Jonasch et al., 2014), and the risk of metastasis and recurrence is also high (Cohen and McGovern, 2005). In addition, the heterogeneity of tumors makes great differences in the survival rate of patients. In recent years, many molecular biomarkers of ccRCC have been found. C1q/tumor necrosis factor (C1QTNF) (Lin W et al., 2020) and 6-snoRNA characteristics (Zhao et al., 2020) can be used as independent prognostic indicators of ccRCC. Due to the lack of accurate and effective ccRCC therapeutic molecular targets, it is still of great significance to explore new prognostic molecular markers or therapeutic targets in ccRCC.

Macrophages are a kind of innate immune cells, which have the functions of chemotaxis, phagocytosis, regulating inflammatory response, and killing microorganisms, and are an important part of the nonspecific immunity of the body. Recently, it has been found that macrophages can change their phenotypes according to the changing microenvironment, and thus have diverse functions (Shapouri-Moghaddam et al., 2018). There are two major macrophage phenotypes, classically activated macrophages (M1) and selectively activated macrophages (M2), which represent the two extremes of macrophage polarization. M1 macrophages are the main effector cells for the host to destroy pathogens, while M2 macrophages can suppress inflammatory responses and promote angiogenesis and tissue remodeling and repair (Sica and Mantovani, 2012). Research indicates that macrophage differentiation is related to tumorigenesis (Cao et al., 2021). M2-polarized tumor-associated macrophages (M2-TAMs) can help tumor cells change the microenvironment; promote tumor growth, angiogenesis, invasion, and metastasis; and inhibit antitumor immune response (Yuan et al., 2020). M2 macrophages play an important role in the progression and infiltration of lung cancer (Pritchard et al., 2020) and gastric and breast cancer (Chen et al., 2017). Wang Y et al. (2021) study showed that M2 macrophages correlated with the immune microenvironment of clear cell renal cell carcinoma. The key genes involved in macrophage differentiation are of great significance for improving the prognosis and immunotherapeutic effect of patients with hepatocellular carcinoma (Cao et al., 2021). Macrophage differentiation–associated genes (MDGs) may be of great value in screening tumor markers and predicting tumor prognosis. However, the prognostic value and immunotherapy of MDGs in patients with ccRCC have not been reported.

This study mainly constructs a prognostic model based on MDGs and establishes a nomogram to predict individual survival probability by combining clinicopathological features and prognostic genes. The Tumor Immune Estimation Resource (TIMER) database was used to analyze the correlation between prognostic genes and immune infiltrating cells. Moreover, the expression levels of the prognostic genes were verified in clinical ccRCC tissues. It provides a basis and new reference for the treatment of ccRCC and also provides a basis for the exploration of molecular mechanisms related to the prognosis of ccRCC.

## Materials and Methods

### Data Source

The RNA-sequencing data set and clinical data of 530 ccRCC and 72 normal tissue samples were downloaded from The Cancer Genome Atlas (TCGA) (https://portal.gdc.cancer.gov/) database. Transcriptomic and clinical information of 91 ccRCC tissue samples was obtained from the International Cancer Genome Consortium (ICGC) database (https://dcc.icgc.org/) as a validation set for the model.

A total of 453 macrophage differentiation–associated genomes (MDGs) were identified using the Gene Cards database (http://www.genecards.org/) and the Gene Set Enrichment Analysis (GSEA) gene set (https://www.gsea-msigdb.org/gsea/msigdb/index.jsp). To verify the robustness of the results, we used the Gene Expression Omnibus (GEO) database to perform a validation of hub gene expression level, among which 72 ccRCC and 72 normal tissue samples were selected in the GSE53757.

### Identification of Differentially Expressed Macrophage Differentiation–Associated Genomes

The “limma” package was used to identify differentially expressed MDGs in the TCGA—ccRCC and normal tissue samples, with |log 2 (fold change, FC)| > 1 and *p* < 0.05 as the cut-off values, and the result was shown as a volcano map.

### Identification of Differentially Expressed Prognosis-Related Macrophage Differentiation–Associated Genomes

In order to obtain the MDGs associated with the prognosis of ccRCC, the univariate Cox regression analysis was performed on 438 MDGs that were present in ccRCC, and *p* < 0.05 was identified as significant. Differentially expressed MDGs and prognosis-related MDGs then were taken to intersect to obtain prognostic differentially expressed MDGs. The STRING (https://string-db.org) website was utilized for building a protein–protein interaction (PPI) network, and the “corrr” package in R was used to construct the correlation network of the obtained prognostic differentially expressed MDGs.

### Construction and Verification of the Prognostic Model

To better predict the outcome of ccRCC patients, we divide the TCGA–ccRCC sample into a training set and test set at a ratio of 6:4. The univariate Cox analysis and the multivariate Cox regression analysis were performed in the TCGA–ccRCC training set, and then the prognostic model was obtained and validated. In brief, we considered key MDGs that are significantly related to prognosis by the univariate Cox analysis as influencing factors. After bringing them into the multivariate Cox proportional hazard model, significant MDGs will be retained during multiple computing. Multiple genes with a *p* value less than 0.05 in the univariate Cox regression analysis were subjected to multivariate Cox analysis. Subsequently, the stepwise regression function was used, and the parameter direction was set as “both” to adjust the multivariate regression model, and finally, the optimal model was obtained. The weighted coefficients based on individual gene expression levels were used to calculate the risk score as follows: risk score = ∑ regression coefficient (genei) × expression value (genei). The patients in the training set were then stratified into the low- and high-risk groups according to median risk score values, and survival was analyzed using the Kaplan–Meier (K-M) analysis. The K-M analysis and log-rank test were used to assess differences in survival between different groups using the package “survival” in R, and the package “survminer” was used for visualizing the results. The 1-, 3-, and 5-year survival was determined by the receiver operating characteristic (ROC) curve analysis using the “survivalROC” package in R, and the areas under the curve (AUC) were calculated. The prognostic model was further validated in the TCGA—ccRCC test set and the ICGC external validation set.

### Association of the Prognostic Model and Clinicopathological Features

To further investigate the prognosis of clinicopathological features and risk score, the correlation between clinical factors including gender, age, stage, grade, T/M/N, and risk score was performed in the TCGA–ccRCC training set first, Wilcoxon test was used to compare differences in risk scores between gender, age, M, and N subgroups, and Kruskal–Wallis test was used to analyze the association between clinicopathological features (stage, grade, and T) and risk scores. Then, the independent prognostic factor was analyzed. In brief, clinical factors including gender, age, stage, grade, T/M/N, and risk scores were determined by univariate Cox analysis. Multivariate Cox regression analysis was then used to identify the independent prognostic factors for ccRCC. Prognostic nomograms were generated using the least absolute shrinkage and selection operator (LASSO) with the “glmnet” package in the TCGA—ccRCC training set that includes gender, age, stage, grade, T/M/N, and risk score clinical characteristics.

### Analysis of the Kyoto Encyclopedia of Genes and Genomes Signal Pathway in High- and Low-Risk Groups

To analyze the signaling pathway differences between high- and low-risk groups, the “gsva” package in R was used to calculate the score of the Kyoto Encyclopedia of Genes and Genomes (KEGG) signal pathway in each sample. The KEGG pathway of the top 50 was plotted on a heat map.

### Analysis of the Correlation Between Prognostic Macrophage Differentiation–Associated Genomes and Tumor-Infiltrating Immune Cells

The correlation analysis between prognostic MDGs and tumor-infiltrating immune cells (including tumor purity, B cells, CD4+T cells, CD8+T cells, macrophages, neutrophils, and dendritic cells) was performed using the TIMER database in ccRCC, and *p* < 0.05 was considered statistically significant. Also, the Spearman correlation analysis was used to detect their correlation.

### Verification of Prognostic Macrophage Differentiation–Associated Genome Expression Level

The mRNA and protein expression level of prognostic MDGs in the GSE53757 datset was verified by using the Wilcoxon test paired test method.

### RNA Extraction and Quantitative Real-Time Polymerase Chain Reaction

The mRNA expression levels of prognostic genes were detected in 10 pairs of ccRCC tissue samples and para-cancerous control tissue samples from the First Hospital of ShanXi Medical University. This study was allowed by the Ethics Committee of the First Hospital of ShanXi Medical University. All patients had approved for the use of clinical tissues for research purposes. Total RNA was isolated using TRIzol (Genecopoeia). The SureScript-First-strand-cDNA-synthesis-kit (Genecopoeia) was used for first-strand cDNA synthesis. For the analysis of the target gene mRNA levels, qPCR was performed using BlazeTaq™ SYBR ^®^ Green qPCR Mix 2.0 according to the manufacturer’s instructions (Genecopoeia). The relative expression of mRNA was calculated by the 2^*−ΔΔCt*^ method with the normalization to GAPDH. All specific primers were shown in Table 1.

**TABLE 1:** Primer sets were used for the PCR assay in this study.

Target gene		Sequence (5′→3′)
KDF1	F	TGT​CGA​GCC​GAG​AAA​TTG​AT
	R	GGT​GGT​AGT​CCT​GCG​TGA​TG
KITLG	F	CTT​GTG​GAG​TGC​GTG​AAA​G
	R	AGG​AGT​AAA​GAG​CCT​GGG​T
TGFA	F	GTA​TTG​TGT​TGG​CTG​CGT​G
	R	CTG​AGT​GTG​GGA​ATC​TGG​G
CD14	F	GCA​ACA​CAG​GAA​TGG​AGA​C
	R	GAA​CGA​CAG​ATT​GAG​GGA​G
ABCG1	F	GGA​TGA​AGG​CAG​AAG​GGA​A
	R	GTG​CAA​ATG​ATG​GAG​CGA​C
HAVCR2	F	CCT​ATC​TGC​CCT​GCT​TCT​AC
	R	CTC​CCC​AGT​GTC​TGT​GTC​TC
GAPDH	F	CCC​ATC​ACC​ATC​TTC​CAG​G
	R	CAT​CAC​GCC​ACA​GTT​TCC​C

## Results

### Identification of Differentially Expressed Prognostic Related Macrophage Differentiation-Associated Genomes

**FIGURE 1:**
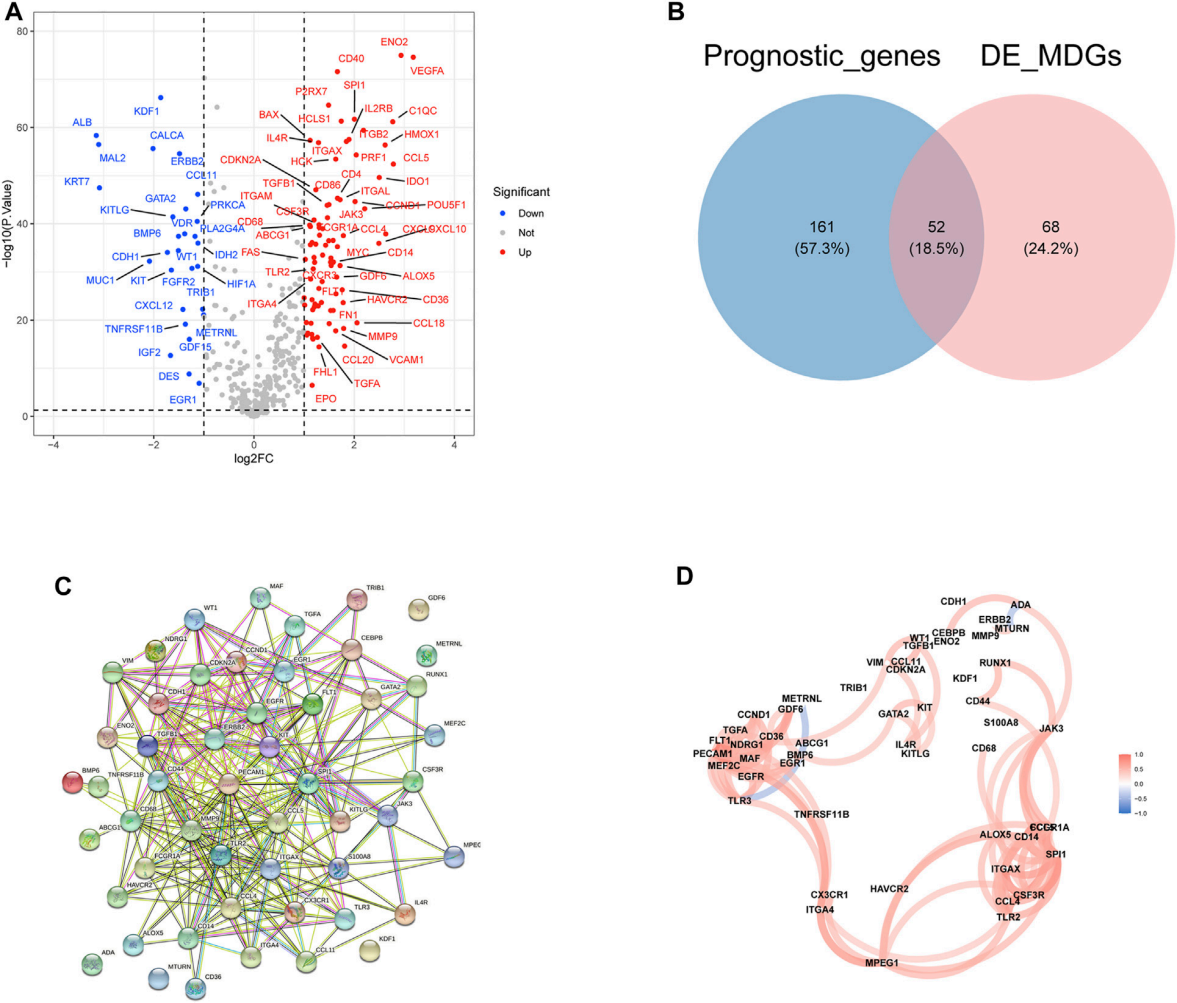
Identification and the differential expression analysis of prognostic related MDGs in clear cell renal cell carcinoma (ccRCC). **(A)** The volcano plot of differential expressed MDGs in TCGA-ccRCC tissues vs. normal tissues. **(B)** The Venn plot of prognostic MDGs and differentially expressed MDGs. **(C)** Protein–protein interactions of the 52 differentially expressed prognosis-related MDGs. **(D)** The correlation analysis of 52 differentially expressed prognosis-related MDGs.

A total of 120 differentially expressed MDGs were selected where 87 MDGs were upregulated and 33 MDGs were downregulated in ccRCC samples (Figure 1A). A total of 438 MDGs in ccRCC were obtained from the Gene Cards database and GSEA gene se and, then the univariate Cox analysis found that 213 MDG were significantly related to the prognosis of ccRCC. Finally, 52 differentially expressed prognostic-related MDGs were obtained by taking the intersection of 120 differentially expressed MDGs and 213 significantly prognostic-related MDGs of ccRCC (Figure 1B). The PPI network analysis of 52 MDGs obtained a protein interaction network comprising 52 nodes and 286 edges (Figure 1C). In addition, further analysis revealed that there was a positive correlation between multiple MDGs (Figure 1D).

### Construction of Prognostic Model in Clear Cell Renal Cell Carcinoma

**FIGURE 2:**
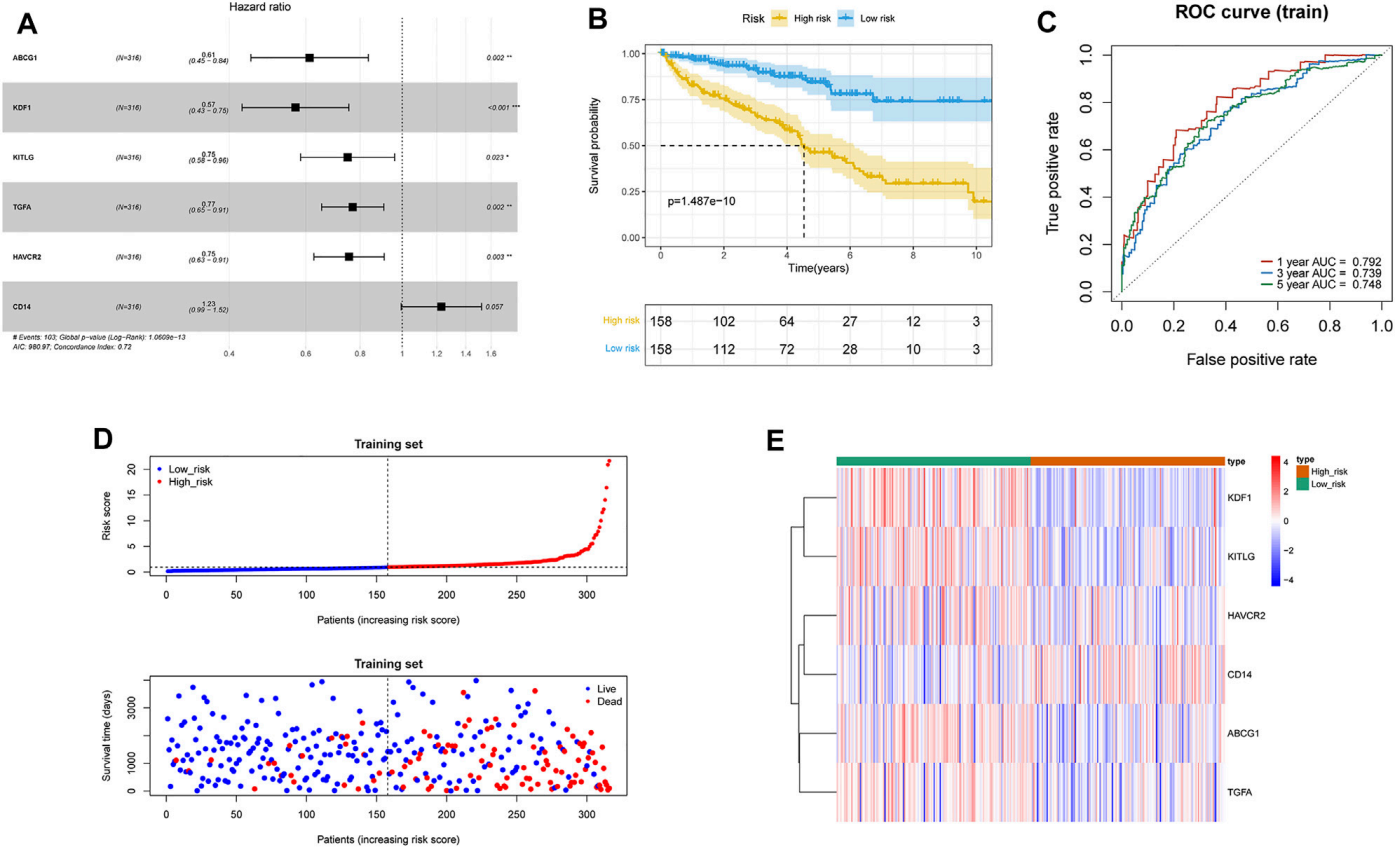
Construction of the macrophage differentiation-related prognostic model in ccRCC. **(A)** Forest plot of MDG Hazard Ratio based on the multivariate Cox regression analysis. **(B)** Survival curves of high- and low-risk groups in the TCGA-ccRCC training set. **(C)** Time-dependent ROC curve of the prognostic model for 1-, 3-, and 5-year overall survival in the training set. **(D)** Distribution of risk score and survival status of the ccRCC patients in the training set, and the dotted line represented the median risk score and divided the patients into low-risk and high-risk groups. **(E)** Heatmap of prognostic gene expression in high- and low-risk groups in the training set.

Thirty-four survival-associated MDGs were identified in the training group by univariate Cox analysis, of which ABCG1, KDF1, PECAM1, ERBB2, KITLG, JAK3, ADA, TGFA, CEBPB, CDKN2A, FLT1, MTURN, TLR3, CCND1, BMP6, CDH1, GDF6, MMP9, KIT, NDRG1, RUNX1, WT1, ITGAX, METRNL, HAVCR2, ENO2, FCGR1A, CSF3R, CD68, CD44, GA TA2, S100A8, TNFRSF11B, and CD14 were further screened by the multivariate Cox proportional hazards model. A six-gene (ABCG1, KDF1, KITLG, TGFA, HAVCR2, CD14) prognostic model was constructed using regression coefficients of each gene, and the risk score was calculated (Figure 2A). The risk scores were calculated for each patient in the training group, and the patients were assigned to the high-risk or low-risk group based on the median risk score. As shown in Figure 2B, patients with high-risk scores had significantly poorer survival outcomes than those with low-risk scores (*p* < 0.05). Furthermore, the AUC of the risk score for 1-year, 3-year, and 5-year overall survival (OS) was 0.792, 0.739, and 0.748, respectively (Figure 2C). The survival status, risk scores, and gene expression data of ccRCC patients in the training group are illustrated in Figure 2D.

### Validation of the Prognostic Model

**FIGURE 3:**
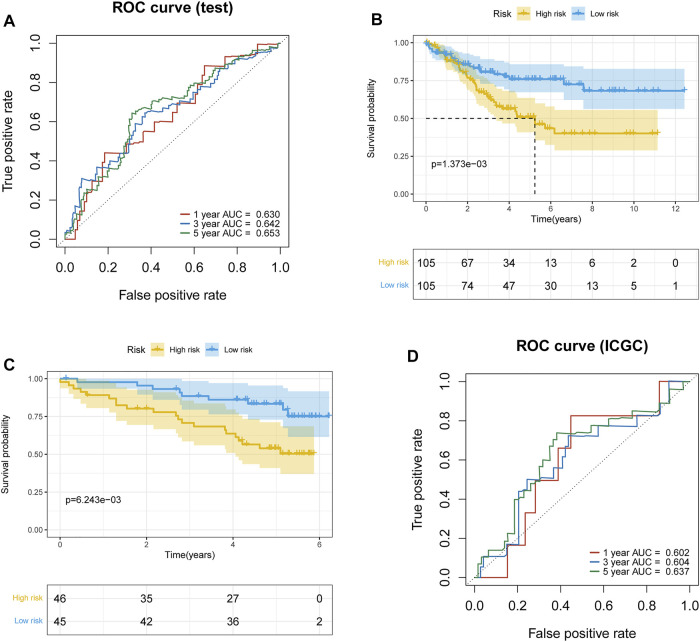
Validation of the prognostic model. **(A)** Time-dependent ROC curve of the prognostic model for 1-, 3-, and 5-year overall survival in the TCGA–ccRCC test set. **(B)** Survival curves of high- and low-risk groups in the TCGA–ccRCC test set. **(C)** Survival curves of high- and low-risk groups in the ICGC test set. **(D)** Time-dependent ROC curve of the prognostic model for 1-, 3-, and 5-year overall survival in the ICGC test set.

To further validate the prognostic signature associated with macrophage differentiation, its prognostic accuracy was further assessed in three independent cohorts, including the ICGC validation group and TCGA–ccRCC test set. The OS was significantly longer for patients in the low-risk than in the high-risk group in the TCGA–ccRCC test set (Figure 3A), and the predicted 1-year, 3-year, and 5-year OS was 0.630, 0.642, and 0.653, respectively (Figure 3B). The ICGC validation cohort also validated the prognostic accuracy of the prognostic model (Figure 3C), with respective AUCs of 0.602, 0.604, and 0.637 for 1-year, 3-year, and 5-year OS, respectively (Figure 3D). Thus, the prognostic model predicted the OS of ccRCC patients with superior accuracy.

### The Prognostic Model Confers Additional Prognostic Power for Clear Cell Renal Cell Carcinoma Patients

**FIGURE 4:**
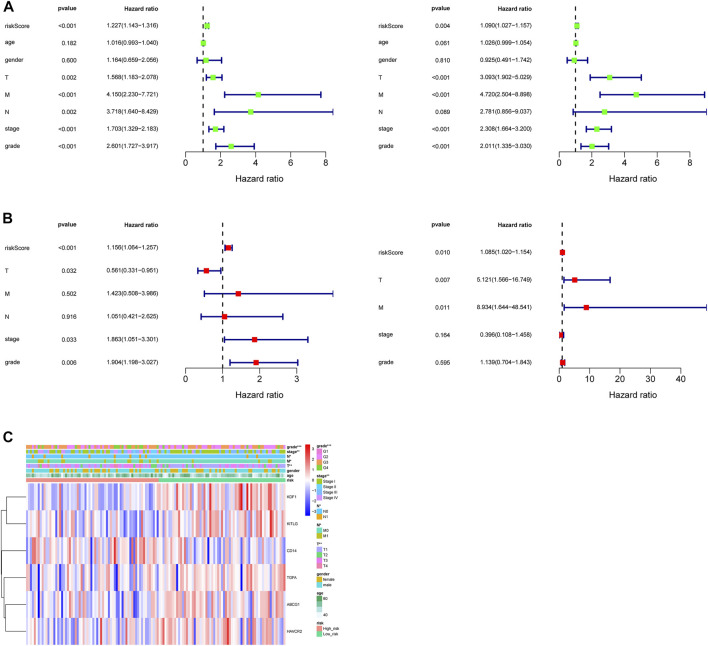
Independent prognostic value of the prognostic model. **(A)** Forest plot of associations between risk factors and the survival of ccRCC based on univariate Cox regression analysis in the training set (left) and test set (right). **(B)** Risk score was an independent predictor of ccRCC based on the multivariate Cox regression analysis in the training set (left) and test set (right). **(C)** Heatmap of the expression profiles of the six prognostic MDGs in different clinicopathological characteristics in the training set.

**FIGURE 5:**
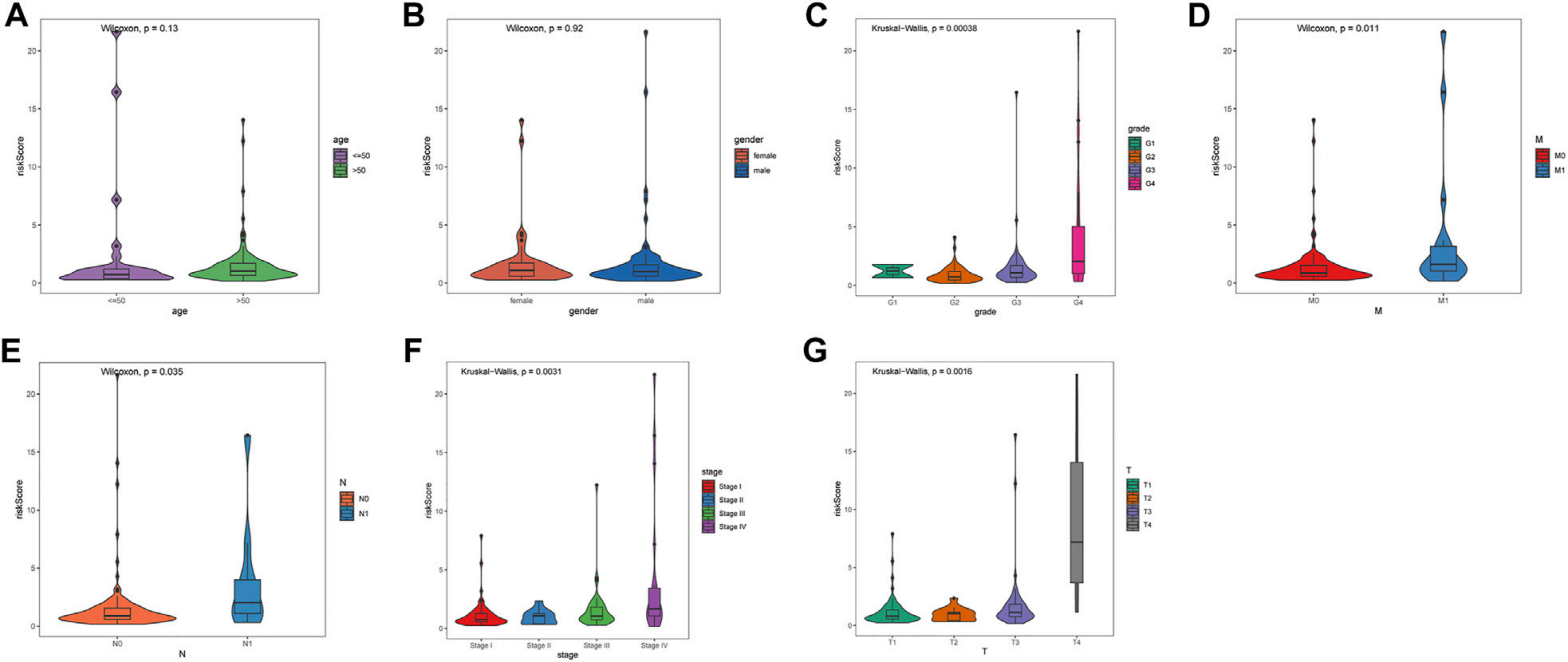
Differences in risk score among seven clinicopathological factors. **(A)** age. **(B)** gender. **(C)** grade. **(D)** pathological M. **(E)** pathological N. **(F)** stage. **(G)** pathological T. Statistical test: Wilcoxon (in two groups) and Kruskal–Wallis (in three or more groups).

Seven clinicopathological factors, including gender, age, stage, grade, and T/M/N, were included in the prognostic model for univariate Cox independent analysis. As shown in Figure 4A, clinical factors including risk score, T/M/N, stage, and grade were closely associated with patient survival. Multivariate Cox regression analysis further showed that the risk score was an independent prognostic indicator for OS in the TCGA–ccRCC cohort (Figure 4B). Expression profiles of the six MDGs are shown in Figure 4C and significant differences were found in the expression among stages, grades, T, M, and N. In addition, the risk score was significantly different in different stages, grades, T, M, and N (Figure 5).

### Construction and Verification of a Nomogram

**FIGURE 6:**
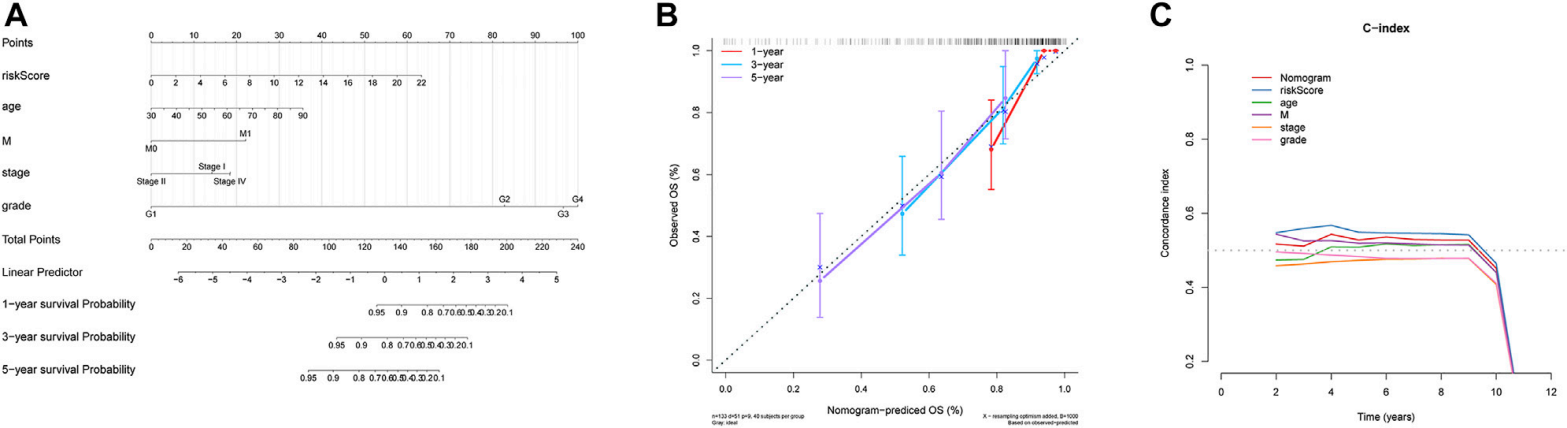
Nomogram can predict the prognosis probability in ccRCC. **(A)** Nomogram to predict the OS possibility of 1 year, 3 year, and 5 year. **(B)** Correction curve of the nomogram. **(C)** Concordance index (C-index) of the nomogram.

In order to establish a clinical method for predicting the survival probability of ccRCC patients, we constructed a nomogram to predict the possibility of 1-year, 3-year, and 5-year OS. As shown in Figure 6A, the score assigned to each factor is proportional to its risk contribution to survival. Thus, based on the patient’s characteristic scores, we could obtain 1-, 3-, and 5-year survival rates for ccRCC patients. We draw a nomogram correction curve based on the aforementioned prediction model. The results show that the prediction accuracy of the model for patient 1-, 3-, and 5-year survival rates is very high, indicating that the constructed prediction model can be used as an effective model (Figure 6B). In addition, the concordance index analysis shows that the risk score, nomogram, and M were all higher than 0.5 of 1–8 years of OS prediction (Figure 6C).

### Differential Analysis of Kyoto Encyclopedia of Genes and Genomes Enrichment in High- and Low-Risk Groups

**FIGURE 7:**
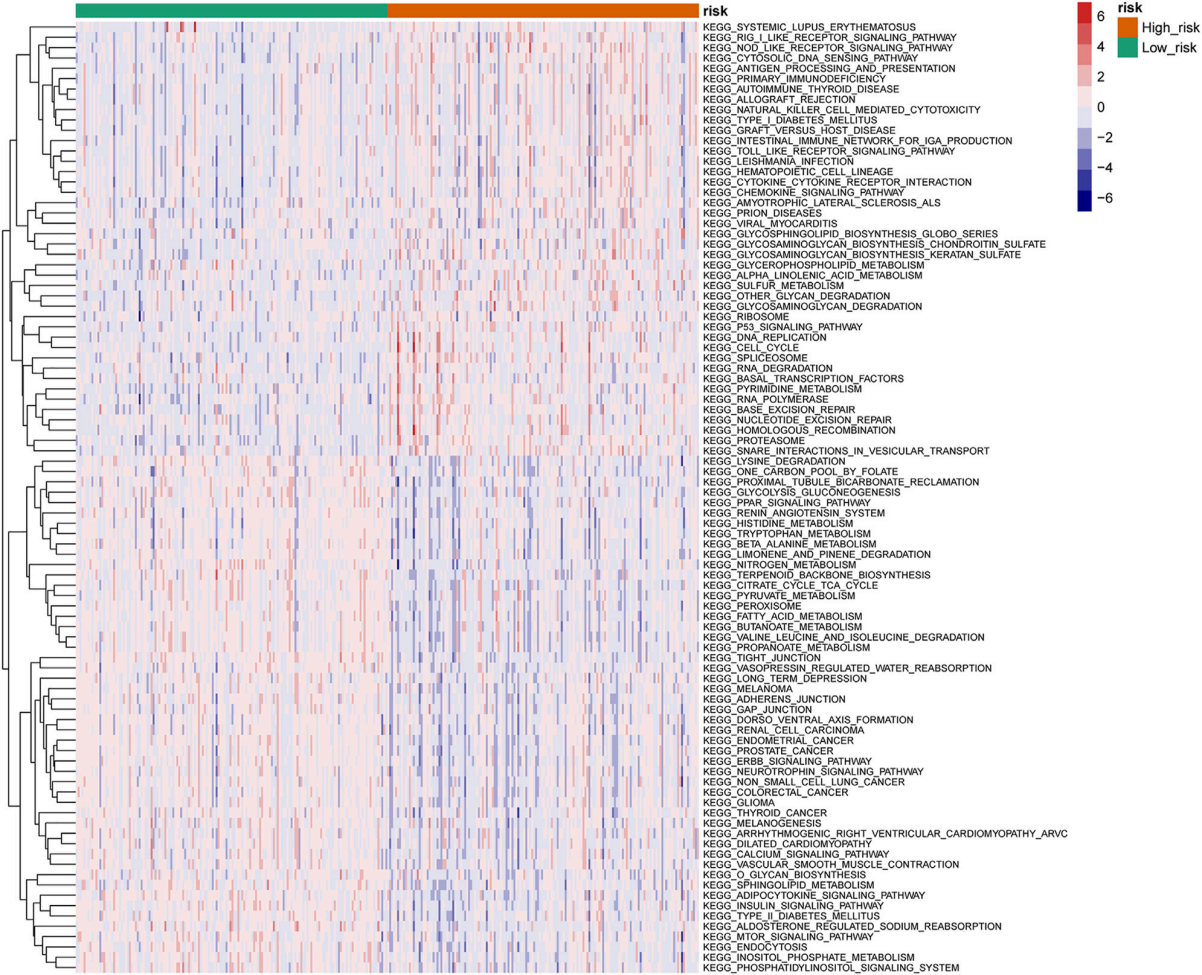
Heatmap of the KEGG pathway in high- and low-risk groups (top 50).

Differential analysis of KEGG pathway enrichment in high- and low-risk groups revealed that the high-risk group was mainly enriched in the systemic lupus erythematosus, RIG l-like receptor signaling pathway, NOD-like receptor signaling pathway, cytoplasmic DNA sensing pathway, antigen processing and presentation, primary immunodeficiency, etc. pathways, while the low-risk group was mainly enriched in the lysine degradation, proximal tubule bicarbonate recovery, glycolytic gluconeogenesis, PPAR signal pathway, renin–angiotensin system, etc. pathways (Figure 7).

### Analysis of the Correlation Between Prognostic Macrophage Differentiation–Associated Genomes and Immune Infiltration

**FIGURE 8:**
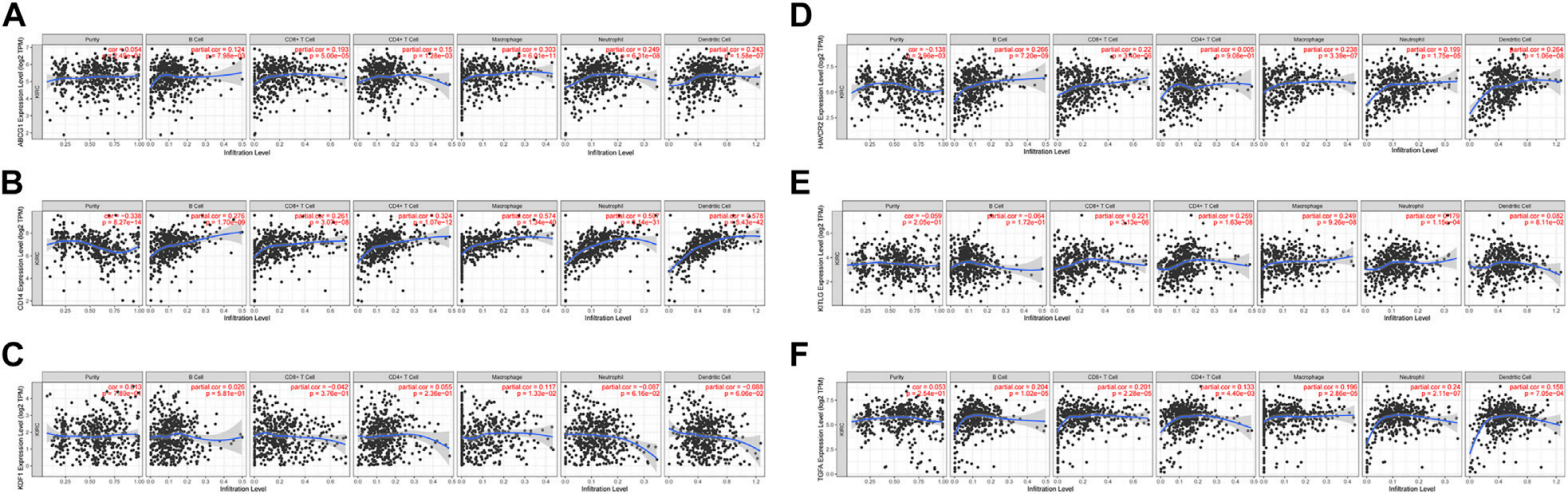
Correlation analysis between the six prognostic genes and infiltration abundances of six types of immune cells (tumor purity, B cells, CD8^+^ T cells, CD4^+^ T cells, macrophages, neutrophils, and dendritic cells). **(A)** ABCG1. **(B)** CD14. **(C)** KDF1. **(D)** HAVCR2. **(E)** KITLG. **(F)** TGFA.

Correlation analysis of six prognostic MDGs (ABCG1, KDF1, KITLG, TGFA, HAVCR2, and CD14) and six types of immune infiltrating cells, including tumor purity, B cells, CD8+T cells, CD4+T cells, macrophages, neutrophils, and dendritic cells (Figures 8A–F) was performed. ABCG1 was significantly correlated with CD8^+^ T cells, macrophages, neutrophils, and dendritic cells. CD14 was significantly correlated with purity, B cells, CD8^+^ T cells, CD4+T cells, macrophages, neutrophils, and dendritic cells. HAVCR2 was significantly correlated with B cells, CD8^+^ T cells, macrophages, neutrophils, and dendritic cells. KITLG was significantly correlated with CD8+T cells, CD4+T cells, macrophages, and neutrophils. TGFA was significantly correlated with B cells, CD8^+^ T cells, macrophages, neutrophils, and dendritic cells.

### Detection and Validation of Prognostic Gene Expression

**FIGURE 9:**
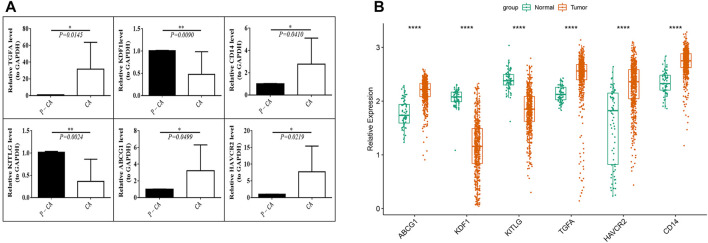
Verification of the prognostic MDG expression level. **(A)** mRNA expression levels of prognostic genes in ccRCC tissue samples (CA) and para-cancerous control tissue samples (P-CA). *p < 0.05, **p < 0.01. **(B)** mRNA expression levels of the six prognostic MDGs in the GSE53757 data set.

We tested the mRNA expression differences of six prognostic genes in 10 pairs of ccRCC tissue samples and para-cancerous control tissue samples. The results found that the expression levels of ABCG1, HAVCR2, CD14, and TGFA were significantly increased (*p* < 0.05) in tumor tissue samples compared with para-cancerous control tissue samples, while the expression levels of KDF1 and KITLG were significantly decreased (*p* < 0.05) (Figure 9A). The same expression trend was seen in the GSE53757 dataset (Figure 9B).

## Discussion

Macrophages can be activated and differentiated into macrophage subtypes with different molecular phenotypes and functional characteristics according to different environments, which are regulated by macrophage differentiation–related genes (MDGs). Activated macrophages included the classically activated (M1) subtype macrophages and the selectively activated (M2) subtype macrophages (Ruytinx et al., 2018). It is found that most macrophages in tumor tissues show the characteristics of M2 macrophages, which suggests that there is a special microenvironment that can promote macrophages mainly toward M2 subtype macrophages in tumor tissues (Wang H et al., 2021). Tumor-associated macrophages (TAMs) are the most abundant inflammatory infiltrates in the tumor microenvironment. It is generally believed that TAMs are mainly M2 macrophages. TAMs can promote tumor growth, invasion, metastasis, and drug resistance (Pan et al., 2020). In renal cell carcinoma, TAMs are associated with tumor invasion, poor prognosis, angiogenesis, and immune escape (Kovaleva et al., 2016; Fu et al., 2019); therefore, MDGs as regulators of macrophage differentiation are probably used as biomarkers of ccRCC and potential prognostic indicators of malignant tumors. Herein, to identify the prognostic value of MDGs associated with ccRCC, first, we identified that MDG sets were significantly enriched in ccRCC tissues compared with paired normal tissues, which is consistent with the previous research results (Gottfried et al., 2003). Subsequently, we identified and validated a prognostic model consisting of six gene combinations for ccRCC. Furthermore, risk scores can strongly predict the ccRCC patient outcomes.

The six MDGs (ABCG1, HAVCR2, CD14, TGFA, KDF1, and KITLG) we identified in this study were significantly associated with ccRCC patient outcomes and therefore may play an important role in disease progression. All these genes have been previously reported to be involved in cancers. ABCG1 (ATP-binding cassette G1) belongs to the ATP-binding cassette (ABC) transporter family, which is involved in lipid balance and cholesterol efflux from macrophages (Vasiliou et al., 2009). It was found to be a potential biomarker for lung cancer (Tian et al., 2017), head and neck squamous cell carcinoma (Gonzalez et al., 2003), prostate cancer (Demidenko et al., 2015), and ccRCC (Meng et al., 2021). HAVCR2, also known as TIM-3 (T-cell immunoglobulin domain and mucin domain-3), is involved in the pathogenesis of malignant tumors and the progression of various types of cancer (Pu et al., 2018). Overexpression of TIM-3/HAVCR2 is associated with poor prognosis in squamous cell carcinoma, colorectal, renal cell carcinoma, gastric, and breast cancers (Shen et al., 2016; Wang et al., 2016; Wang et al., 2017; Stenzel, 2018). CD14, a kind of glycoprotein located on the cell surface, was derived mostly from monocytes, macrophages, and neutrophil granulocytes, which mainly acts as a receptor of lipopolysaccharides (LPSs) (Wright et al., 1990). Studies have shown that the CD14 gene is related to macrophage differentiation and regulates immune cell activation (Stenzel, 2018) and is closely related to tumor cell proliferation and the tumor microenvironment in cancers (Wang et al., 2014; Cheah et al., 2015; Guan et al., 2020). TGFA, (transforming growth factor A) as a member of the epidermal growth factor family, is encoded by the TGFA gene. A previous study showed that TGFA is involved in the differentiation of tumor-associated macrophages (TAMs) (Gupta et al., 2018). TGFA is highly expressed in tumor tissues such as bladder cancer, oral cancer, pancreatic cancer, and small cell lung cancer, accelerating cell proliferation, invasion, and EMT (epithelial–mesenchymal transition), and is closely related to the prognosis of tumor patients (Kang et al., 2005; Liu et al., 2017; Hao et al., 2018; Zhang et al., 2021). KDF1 (Keratinocyte Differentiation Factor 1) was first reported as a key role in the development of normal epidermis by regulating the proliferation and differentiation of keratinocytes (Lee et al., 2013). Research showed that KDF1 was decreasingly expressed in the cancer cells and correlated negatively with the tumor grade and positively with the survival of the patients, which means KDF1 may function as a tumor suppressor (Zheng et al., 2021). Our study showed that the expression of KDF1 decreased in ccRCC. KITLG is also known as stem cell factor, steel factor, and mast cell growth factor, whose aberrant expression has been implicated in the development of several cancers (Yang et al., 2014; Yang et al., 2015). Our study suggests a decreased expression of KITLG in ccRCC which means KITLG may be a protective gene for ccRCC.

In order to clarify the potential rational mechanism of this prognostic model, we performed GSEA analysis to identify the enriched biological process and pathway in the high-risk cohort compared with the low-risk cohort. Interestingly, the analysis shows that the high-risk group was mainly enriched in the systemic lupus erythematosus. Systemic lupus erythematosus and ccRCC seem to be diseases of distinct patterns. However, there are hidden connections between cancer and systemic lupus erythematosus, which involve multiple of metabolic routes. A previous study showed that systemic lupus erythematosus is highly associated with increased cancer risk compared with the general population (Song et al., 2018). The underlying pathophysiologic mechanisms are still not fully understood, but possible factors include lupus-related medications, inherent immune system abnormalities, viral infections, and/or traditional cancer risk factors (Ladouceur et al., 2020). Other pathways which significantly enriched in a high-risk cohort include the RIG l (retinoic acid inducible-gene 1)-like receptor signaling pathway, the NOD (nucleotide-binding oligomerization domain)-like receptor signaling pathway, the cytoplasmic DNA sensing pathway, antigen processing and presentation, primary immunodeficiency, etc. RIG 1 is the pattern recognition receptor in the cytoplasm that can activate Type I interferon to trigger an antiviral immune response (Ranoa et al., 2016), and Type I IFNs are involved in cancer immunosurveillance and anticancer immune responses widely (Zhang F et al., 2020). NOD-like receptors represent a class of widespread, sophisticated signaling regulators and have been established as crucial regulators in inflammation-associated tumorigenesis, angiogenesis, cancer cell stemness, and chemoresistance (Liu et al., 2019). The cytoplasmic DNA sensing pathway plays an important role in antitumor immunity as well as cancer progression, genomic instability, and the tumor microenvironment (Kwon and Bakhoum, 2020). These aforementioned signaling pathways, including antigen processing and presentation, are all closely related to tumor immunity, which means the immune escape of tumor cells and the change of tumor immune microenvironment are important mechanisms of ccRCC. The low-risk group was more associated with lysine degradation, proximal tubule bicarbonate recovery, glycolytic gluconeogenesis, PPAR signal pathway, renin–angiotensin system, etc. Therefore, we speculate that the competition between carcinogenic factors and body resistibility results in disturbed metabolic microenvironments such as amino acids and glucose metabolism and hydroelectrolyte balance. The low-risk group might benefit more from metabolism-related treatment than the high-risk group. These hypotheses need further investigation.

Immune infiltration of tumors is closely associated with clinical outcomes in renal cell carcinoma, tumor-infiltrating immune cells (TIICs) have independent prognostic values in RCC (Zhang et al., 2019). In order to further understand the relationship between these six prognostic MDGs and tumor immunity of ccRCC, we made a correlation analysis between these prognostic MDGs and tumor immune infiltrating cells. The results showed a significant correlation between these six prognostic MDGs and the immune infiltrating cells including CD8^+^ T cells, CD4^+^ T cells, macrophages, neutrophils, and dendritic cells. At present, PD-1/PD-L1 immune inhibitor is the first-line treatment of metastatic ccRCC. Despite the encouraging activity and tolerable toxicity of PD-1/PD-L1, there is the additional risk of immune-mediated side effects (Grimm et al., 2020), and its diagnosis and prognosis still lack reliable biomarkers. Therefore, many studies were devoted to finding biomarkers that can predict the response of these therapies. Several studies have found some prognostic-related biomarkers such as CD8 (+) T lymphocytes (Zhang et al., 2019; Lin J et al., 2020) and tumor-infiltrating CD19 (+) B lymphocytes (Lin et al., 2018); however, a single biomarker is not enough to identify the efficacy of immunotherapy accurately. The prognostic model we established by the expression of the six MDGs which showed high accuracy in predicting the 1-, 3-, and 5-year survival rate of ccRCC patients suggested that the prognostic model may be helpful in developing biomarkers and performing prognostic evaluation of metastatic clear cell renal cell carcinoma.

Moreover, we established a nomogram for clinical-decision support. Owing to their intuitive visual presentation and personalized application, nomograms have become a popular tool for oncology prognosis these years. Zhang Z et al. (2020) reported a nomogram based on five deferentially expressed genes to predict 1-, 3- and 5-year OS in ccRCC patients. Peng et al. (2021) constructed a nomogram based on the dysregulated ceRNA network and hope to find a better understanding of the ccRCC tumorigenesis mechanism. The nomogram we established incorporates age, tumor staging, grading, tumor metastasis, and risk score based on clinicopathological factors. By calculating the score of each risk factor, the numerical outcomes are finally generated for the individual patients, and the 1-, 3-, and 5-year possibility survival rate can be predicted. The nomogram correction curve and the concordance index analysis both showed excellent accuracy in our nomogram, which means our nomogram could potentially be used in clinical practice for predicting the individual survival rate and promoting the selection of individual treatment options for ccRCC patients.

However, there are still some limitations to our study. First, our study only focused on the macrophage differentiation–associated genes from TCGA and ICGC platforms. Potential prognostic factors, such as environmental factors, genetic factors, and personal history, are missing in our nomogram. Second, we only tested 10 pairs of ccRCC tissue samples and para-cancerous control tissue samples, and more large-scale molecular experiments are needed to further clarify the mRNA expression of six prognostic genes. Third, our study provides the evidence that the six genes could be potential biomarkers in ccRCC and possibly become therapeutic targets for precision medicine in the future, and the exact mechanism of the six genes has not been explored in our study. Therefore, the further functional experiment of the six genes in ccRCC based on larger sample size is still valuable and crucial.

## Conclusion

We developed and validated a six-gene prognostic risk model (ABCG1, HAVCR2, CD14, TGFA, KDF1, and KITLG) associated with macrophage differentiation for ccRCC patients. The higher the risk score, the worse the prognosis. Also, the established nomogram can be used as a novel tool for predicting the clinical outcome of ccRCC patients. However, the model still needs to be validated in prospective clinical trials with large sample sizes in the future.

## Data Availability

Publicly available datasets were analyzed in this study. These data can be found here: https://portal.gdc.cancer.gov/ https://dcc.icgc.org/ https://www.ncbi.nlm.nih.gov/geo/query/acc.cgi?acc=GSE53757.
